# Extracting Cognitive SLUMS Scores from Unstructured National Veterans Clinical Notes with AI

**DOI:** 10.1093/geroni/igaf122.3768

**Published:** 2025-12-31

**Authors:** Rui Ouyang, Christine Rizk, Amir Sharafkhaneh, Sanam Sharafkhaneh, Jose Rios-Monterrosa, Dashiell Helmer, Javad Razjouyan

**Affiliations:** Boston Veterans Healthcare Administration, Boston, Massachusetts, United States; Michael E. DeBakey VA Medical Center, Houston, Texas, United States; Baylor College of Medicine, Houston, Texas, United States; Baylor College of Medicine, Houston, Texas, United States; Baylor College of Medicine, Houston, Texas, United States; Baylor College of Medicine, Houston, Texas, United States; Baylor College of Medicine, Houston, Texas, United States

## Abstract

Clinical assessment of cognitive status is critical to evaluating patient health risk and outcomes yet rarely found within the structured data in the electronic health record (EHR). The Saint Louis University Mental Status (SLUMS) Examination is a cognitive screening tool widely used within the Veterans Affairs (VA). Compared to the Mini-Mental State Examination (MMSE), the SLUMS score has greater sensitivity to earlier stages of cognitive impairment. We present a natural language processing (NLP) method for extracting SLUMS scores from unstructured EHR notes. We identified clinical notes from VA patients that contained the word “SLUMS” and a number within a 500 character window. Two researchers independently annotated 1,275 notes. Of these, 899 contained a single SLUMS score and 376 contained missing, multiple, invalid (typo), or qualitative scores. We developed a rule-based system incorporating regular–expression–based pattern matching. The algorithm was developed on the full set of notes and optimized for precision over recall (i.e., only makes predictions when confident). It achieved 83.0% accuracy and 99.6% precision with an F1 score of 78.2%. We further ran the algorithm on 2.96 million unannotated notes. The algorithm throughput exceeded 20 thousand notes per minute when run on a single laptop processor. This work demonstrates how NLP can extract SLUMS scores with high accuracy at scale from millions of unstructured clinical notes. By creating structured cognitive assessment data from information previously buried in free-form clinical text, our work enables cognitive impairment research at the population scale.

